# 4,4′-Dinitro-2,2′-[propane-1,3-diylbis(iminiumylmethanylyl­idene)]diphenolate

**DOI:** 10.1107/S1600536812015504

**Published:** 2012-04-18

**Authors:** Kwang Ha

**Affiliations:** a School of Applied Chemical Engineering, The Research Institute of Catalysis, Chonnam National University, Gwangju 500-757, Republic of Korea

## Abstract

The title compound, C_17_H_16_N_4_O_6_, is a Schiff base, which is found as a bis-zwitterion in the solid state. The geometry around the iminium N atom indicates *sp* ^2^-hybridization. The diiminiumpropyl­ene chain is in an approximate double-*gauche* conformation, with average N—C—C—C torsion angles of 69.3°. The zwitterion shows strong intra­molecular N—H⋯O hydrogen bonds between the iminium N and phenolate O atom. In the crystal, bifurcated N—H⋯(O,O) hydrogen bonds assemble pairs of molecules into inversion dimers.

## Related literature

For the crystal structure of the related zwitterion, 4-nitro-2-{[(tricyclo­[3.3.1.1^3,7^]decan-1-yl)iminium­yl]meth­yl}phenolate, see: Ha (2012 ▶).
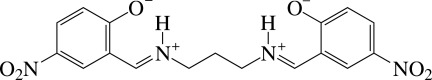


## Experimental

### 

#### Crystal data

C_17_H_16_N_4_O_6_
*M* *_r_* = 372.34
Orthorhombic, 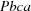

*a* = 11.5698 (5) Å
*b* = 13.0393 (6) Å
*c* = 22.0393 (10) Å
*V* = 3324.9 (3) Å^3^
*Z* = 8
Mo *K*α radiation
μ = 0.12 mm^−1^
*T* = 200 K
0.31 × 0.17 × 0.15 mm

#### Data collection

Bruker SMART 1000 CCD diffractometer
Absorption correction: multi-scan (*SADABS*; Bruker, 2000 ▶) *T*_min_ = 0.890, *T*_max_ = 1.000
23432 measured reflections
4120 independent reflections
2286 reflections with *I* > 2σ(*I*)
*R*_int_ = 0.084

#### Refinement

*R*[*F* ^2^ > 2σ(*F* ^2^)] = 0.052
*wR*(*F* ^2^) = 0.138
*S* = 1.03
4120 reflections
252 parameters
H atoms treated by a mixture of independent and constrained refinement
Δρ_max_ = 0.28 e Å^−3^
Δρ_min_ = −0.30 e Å^−3^

### 

Data collection: *SMART* (Bruker, 2000 ▶); cell refinement: *SAINT* (Bruker, 2000 ▶); data reduction: *SAINT*; program(s) used to solve structure: *SHELXS97* (Sheldrick, 2008 ▶); program(s) used to refine structure: *SHELXL97* (Sheldrick, 2008 ▶); molecular graphics: *ORTEP-3* (Farrugia, 1997 ▶) and *PLATON* (Spek, 2009 ▶); software used to prepare material for publication: *SHELXL97*.

## Supplementary Material

Crystal structure: contains datablock(s) global, I. DOI: 10.1107/S1600536812015504/zl2472sup1.cif

Structure factors: contains datablock(s) I. DOI: 10.1107/S1600536812015504/zl2472Isup2.hkl

Additional supplementary materials:  crystallographic information; 3D view; checkCIF report

## Figures and Tables

**Table 1:** Hydrogen-bond geometry (Å, °)

*D*—H⋯*A*	*D*—H	H⋯*A*	*D*⋯*A*	*D*—H⋯*A*
N2—H3*N*⋯O1	0.93 (3)	1.77 (3)	2.583 (2)	145 (3)
N3—H2*N*⋯O4	0.90 (3)	1.93 (3)	2.642 (2)	135 (2)
N3—H2*N*⋯O4^i^	0.90 (3)	2.17 (3)	2.891 (2)	136 (2)

Symmetry code: (i) 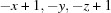.

**Fig. 1.**
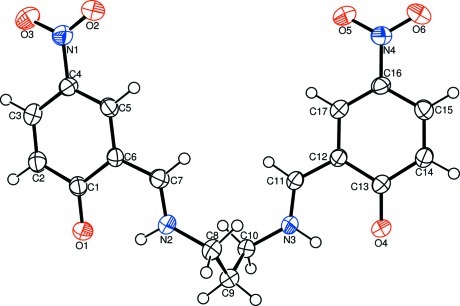
The molecular structure of the title compound, with atom numbering. Displacement ellipsoids are drawn at the 50% probability level for non-H atoms.

**Fig. 2.**
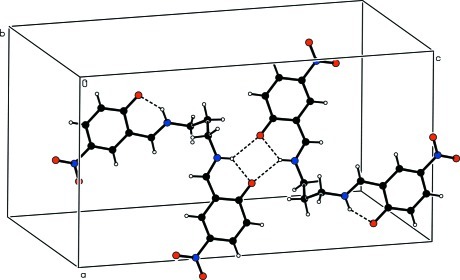
A partial view along the b axis of the crystal packing of the title compound. Intra- and intermolecular N—H···O hydrogen-bonds are shown as dashed lines (see Table 1 for details).

## supplementary crystallographic information

### Comment

The title compound, C_17_H_16_N_4_O_6_, is a tetradentate Schiff base, which can act as a dibasic ligand, that is, the N_2_O_2_ donor atoms can coordinate to a metal ion. In the crystal structure, the Schiff base is found as a bis(zwitterion) in the phenolate-iminium forms (Fig. 1), similar to the structure of the related zwitterion 4-nitro-2-{[(tricyclo[3.3.1.1^3,7^]decan-1-yl)iminiumyl]methyl}phenolate (Ha, 2012). It seems that the acid strength of the phenol was reinforced by the inductive effects of the electron-withdrawing NO_2_ group.

The N—C bond lengths and the C—N—C bond angles indicate that the iminium N atoms are *sp*^2^-hybridized [N2═C7 = 1.292 (2) Å, N2—C8 = 1.456 (3) Å, <C7—N2—C8 = 125.25 (19)°; N3═C11 = 1.293 (3) Å, N3—C10 = 1.457 (3) Å, <C11—N3—C10 = 124.97 (18)°]. The dihedral angles for the four atoms within the diiminiumpropylene chain display that the chain is approximately in the double *gauche* conformation [<N2—C8—C9—C10 = 69.6 (2)° and <C8—C9—C10—N3 = 69.0 (2)°]. The zwitterion shows strong intramolecular N—H···O hydrogen bonds between the iminium N atom and the phenolate O atom, with N···O distances of 2.583 (2) Å and 2.642 (2) Å, forming nearly planar six-membered rings (Fig. 2, Table 1). Two ions are assembled by additional intermolecular N—H···O hydrogen bonds with N···O = 2.891 (2) Å, forming a dimer-type species (Fig. 2, Table 1). In the crystal structure, the benzene rings are not parallel, the dihedral angle between the rings being 11.45 (2)°. Several π-π interactions between the adjacent benzene rings are present, the shortest centroid-centroid distance being 3.5432 (11) Å.

### Experimental

1,3-Diaminopropane (0.3704 g, 5.00 mmol) and 5-nitrosalicylaldehyde (1.6719 g, 10.00 mmol) in EtOH (20 ml) were stirred for 2 h at room temparature. After addition of pentane (30 ml) to the reaction mixture, the formed precipitate was separated by filtration, washed with ether (50 ml), and dried at 323 K, to give a yellow powder (1.8169 g). Yellow block-like crystals, suitable for X-ray analysis, were obtained by slow evaporation of a CH_3_CN solution at room temparature.

### Refinement

The iminium H atoms were located from a difference Fourier map and refined freely. C-bound H atoms were included in calculated positions and treated as riding atoms: C—H = 0.95 Å (CH) or 0.99 Å (CH_2_) with *U*_iso_(H) = 1.2*U*_eq_(C). The highest peak (0.28 e Å^-3^) and the deepest hole (-0.30 e Å^-3^) in the difference Fourier map are located 1.60 Å and 0.81 Å, respectively, from the atoms O3 and H3N.

### Crystal data

**Table:**

C_17_H_16_N_4_O_6_	*F*(000) = 1552
*M**_r_* = 372.34	*D*_x_ = 1.488 Mg m^−^^3^
Orthorhombic, *P**b**c**a*	Mo *K*α radiation, λ = 0.71073 Å
Hall symbol: -P 2ac 2ab	Cell parameters from 3572 reflections
*a* = 11.5698 (5) Å	θ = 2.5–25.3°
*b* = 13.0393 (6) Å	µ = 0.12 mm^−^^1^
*c* = 22.0393 (10) Å	*T* = 200 K
*V* = 3324.9 (3) Å^3^	Block, yellow
*Z* = 8	0.31 × 0.17 × 0.15 mm

### Data collection

**Table:**

Bruker SMART 1000 CCD diffractometer	4120 independent reflections
Radiation source: fine-focus sealed tube	2286 reflections with *I* > 2σ(*I*)
Graphite monochromator	*R*_int_ = 0.084
φ and ω scans	θ_max_ = 28.3°, θ_min_ = 2.5°
Absorption correction: multi-scan (*SADABS*; Bruker, 2000)	*h* = −15→15
*T*_min_ = 0.890, *T*_max_ = 1.000	*k* = −14→17
23432 measured reflections	*l* = −28→29

### Refinement

**Table:**

Refinement on *F*^2^	Primary atom site location: structure-invariant direct methods
Least-squares matrix: full	Secondary atom site location: difference Fourier map
*R*[*F*^2^ > 2σ(*F*^2^)] = 0.052	Hydrogen site location: inferred from neighbouring sites
*wR*(*F*^2^) = 0.138	H atoms treated by a mixture of independent and constrained refinement
*S* = 1.03	*w* = 1/[σ^2^(*F*_o_^2^) + (0.0572*P*)^2^ + 0.0212*P*] where *P* = (*F*_o_^2^ + 2*F*_c_^2^)/3
4120 reflections	(Δ/σ)_max_ < 0.001
252 parameters	Δρ_max_ = 0.28 e Å^−^^3^
0 restraints	Δρ_min_ = −0.30 e Å^−^^3^

### Special details

**Table:**

Geometry. All e.s.d.'s (except the e.s.d. in the dihedral angle between two l.s. planes) are estimated using the full covariance matrix. The cell e.s.d.'s are taken into account individually in the estimation of e.s.d.'s in distances, angles and torsion angles; correlations between e.s.d.'s in cell parameters are only used when they are defined by crystal symmetry. An approximate (isotropic) treatment of cell e.s.d.'s is used for estimating e.s.d.'s involving l.s. planes.
Refinement. Refinement of *F*^2^ against ALL reflections. The weighted *R*-factor *wR* and goodness of fit *S* are based on *F*^2^, conventional *R*-factors *R* are based on *F*, with *F* set to zero for negative *F*^2^. The threshold expression of *F*^2^ > σ(*F*^2^) is used only for calculating *R*-factors(gt) *etc*. and is not relevant to the choice of reflections for refinement. *R*-factors based on *F*^2^ are statistically about twice as large as those based on *F*, and *R*- factors based on ALL data will be even larger.

### Fractional atomic coordinates and isotropic or equivalent isotropic displacement parameters (Å^2^)

**Table:**

	*x*	*y*	*z*	*U*_iso_*/*U*_eq_	
O1	0.18305 (12)	0.22185 (11)	0.21082 (6)	0.0397 (4)	
O2	0.63010 (14)	0.28176 (13)	0.05617 (7)	0.0495 (4)	
O3	0.50263 (15)	0.25797 (13)	−0.01460 (7)	0.0522 (5)	
O4	0.62220 (12)	−0.00556 (11)	0.48734 (6)	0.0383 (4)	
O5	0.96696 (14)	−0.02327 (15)	0.27155 (8)	0.0636 (5)	
O6	1.08562 (13)	−0.02659 (13)	0.34711 (8)	0.0536 (5)	
N1	0.52991 (16)	0.26527 (13)	0.03929 (9)	0.0372 (4)	
N2	0.34001 (16)	0.22765 (13)	0.29410 (8)	0.0325 (4)	
H2N	0.491 (2)	0.0128 (18)	0.4342 (13)	0.061 (8)*	
N3	0.48198 (14)	0.01663 (13)	0.39362 (9)	0.0300 (4)	
H3N	0.266 (3)	0.2203 (19)	0.2781 (13)	0.079 (9)*	
N4	0.98619 (16)	−0.02418 (14)	0.32658 (9)	0.0388 (5)	
C1	0.26324 (17)	0.23326 (14)	0.17156 (9)	0.0293 (5)	
C2	0.23969 (18)	0.23465 (14)	0.10807 (9)	0.0321 (5)	
H2	0.1619	0.2290	0.0947	0.038*	
C3	0.32499 (18)	0.24381 (14)	0.06613 (9)	0.0320 (5)	
H3	0.3065	0.2433	0.0241	0.038*	
C4	0.44063 (18)	0.25404 (14)	0.08474 (9)	0.0296 (5)	
C5	0.46937 (17)	0.25358 (14)	0.14490 (9)	0.0294 (5)	
H5	0.5479	0.2608	0.1567	0.035*	
C6	0.38325 (17)	0.24253 (14)	0.18915 (9)	0.0270 (4)	
C7	0.41546 (18)	0.23887 (14)	0.25139 (9)	0.0305 (5)	
H7	0.4948	0.2449	0.2619	0.037*	
C8	0.36602 (19)	0.22306 (16)	0.35868 (9)	0.0349 (5)	
H8A	0.3365	0.2858	0.3787	0.042*	
H8B	0.4508	0.2208	0.3644	0.042*	
C9	0.31172 (18)	0.12934 (15)	0.38808 (9)	0.0330 (5)	
H9A	0.2285	0.1280	0.3777	0.040*	
H9B	0.3179	0.1364	0.4327	0.040*	
C10	0.36533 (17)	0.02741 (15)	0.36953 (9)	0.0320 (5)	
H10A	0.3166	−0.0295	0.3847	0.038*	
H10B	0.3678	0.0229	0.3247	0.038*	
C11	0.57442 (17)	0.00744 (14)	0.36110 (9)	0.0274 (4)	
H11	0.5665	0.0098	0.3182	0.033*	
C12	0.68702 (17)	−0.00597 (14)	0.38544 (8)	0.0261 (4)	
C13	0.70501 (18)	−0.01066 (14)	0.45046 (9)	0.0281 (4)	
C14	0.82265 (18)	−0.02299 (16)	0.47050 (9)	0.0353 (5)	
H14	0.8382	−0.0277	0.5127	0.042*	
C15	0.91170 (18)	−0.02810 (16)	0.43079 (9)	0.0347 (5)	
H15	0.9885	−0.0358	0.4453	0.042*	
C16	0.89073 (17)	−0.02195 (15)	0.36768 (9)	0.0293 (4)	
C17	0.78028 (17)	−0.01169 (14)	0.34546 (9)	0.0293 (5)	
H17	0.7675	−0.0085	0.3029	0.035*	

### Atomic displacement parameters (Å^2^)

**Table:**

	*U*^11^	*U*^22^	*U*^33^	*U*^12^	*U*^13^	*U*^23^
O1	0.0255 (8)	0.0574 (10)	0.0362 (9)	−0.0014 (7)	0.0025 (7)	0.0090 (7)
O2	0.0335 (9)	0.0701 (12)	0.0450 (10)	0.0044 (8)	0.0064 (8)	0.0105 (8)
O3	0.0570 (11)	0.0726 (12)	0.0269 (9)	0.0072 (8)	0.0058 (8)	−0.0012 (7)
O4	0.0334 (8)	0.0565 (10)	0.0250 (8)	0.0030 (7)	0.0042 (7)	0.0031 (6)
O5	0.0469 (11)	0.1112 (16)	0.0326 (10)	0.0119 (10)	0.0109 (8)	0.0110 (9)
O6	0.0278 (9)	0.0770 (13)	0.0560 (11)	0.0016 (8)	0.0043 (8)	0.0064 (9)
N1	0.0380 (11)	0.0407 (11)	0.0330 (11)	0.0072 (8)	0.0040 (9)	0.0031 (8)
N2	0.0311 (10)	0.0420 (11)	0.0243 (10)	0.0018 (8)	−0.0008 (8)	0.0051 (7)
N3	0.0304 (10)	0.0370 (10)	0.0225 (10)	0.0030 (7)	−0.0016 (8)	−0.0009 (7)
N4	0.0323 (11)	0.0474 (12)	0.0367 (11)	0.0026 (8)	0.0050 (9)	0.0073 (8)
C1	0.0265 (11)	0.0295 (11)	0.0318 (11)	−0.0006 (8)	−0.0022 (9)	0.0047 (8)
C2	0.0304 (11)	0.0321 (12)	0.0336 (12)	−0.0020 (9)	−0.0048 (9)	0.0041 (9)
C3	0.0391 (13)	0.0284 (11)	0.0285 (11)	−0.0002 (9)	−0.0043 (9)	0.0018 (8)
C4	0.0306 (12)	0.0312 (11)	0.0271 (11)	0.0023 (9)	0.0029 (9)	0.0029 (8)
C5	0.0241 (10)	0.0316 (12)	0.0324 (12)	0.0015 (8)	−0.0017 (9)	0.0024 (8)
C6	0.0260 (10)	0.0279 (11)	0.0272 (11)	0.0023 (8)	−0.0019 (9)	0.0040 (8)
C7	0.0285 (11)	0.0314 (11)	0.0315 (11)	0.0009 (9)	0.0011 (9)	0.0022 (8)
C8	0.0368 (12)	0.0418 (13)	0.0260 (12)	0.0022 (10)	−0.0015 (9)	0.0002 (9)
C9	0.0315 (12)	0.0400 (13)	0.0274 (11)	0.0029 (9)	0.0019 (9)	0.0022 (9)
C10	0.0278 (11)	0.0425 (13)	0.0256 (11)	0.0015 (9)	−0.0017 (9)	0.0013 (9)
C11	0.0324 (11)	0.0280 (11)	0.0218 (10)	0.0010 (8)	−0.0012 (9)	−0.0004 (8)
C12	0.0288 (11)	0.0263 (11)	0.0233 (10)	0.0014 (8)	−0.0002 (8)	0.0009 (7)
C13	0.0317 (11)	0.0276 (11)	0.0251 (11)	−0.0018 (8)	0.0004 (9)	0.0010 (8)
C14	0.0331 (12)	0.0469 (13)	0.0260 (11)	−0.0042 (10)	−0.0033 (9)	0.0041 (9)
C15	0.0284 (11)	0.0414 (13)	0.0344 (13)	−0.0037 (10)	−0.0029 (9)	0.0040 (9)
C16	0.0300 (11)	0.0306 (11)	0.0274 (11)	−0.0010 (8)	0.0036 (9)	0.0036 (8)
C17	0.0346 (12)	0.0297 (11)	0.0236 (10)	−0.0003 (9)	0.0011 (9)	0.0015 (8)

### Geometric parameters (Å, º)

**Table:**

O1—C1	1.277 (2)	C5—H5	0.9500
O2—N1	1.236 (2)	C6—C7	1.422 (3)
O3—N1	1.233 (2)	C7—H7	0.9500
O4—C13	1.258 (2)	C8—C9	1.519 (3)
O5—N4	1.233 (2)	C8—H8A	0.9900
O6—N4	1.237 (2)	C8—H8B	0.9900
N1—C4	1.446 (3)	C9—C10	1.523 (3)
N2—C7	1.292 (2)	C9—H9A	0.9900
N2—C8	1.456 (3)	C9—H9B	0.9900
N2—H3N	0.93 (3)	C10—H10A	0.9900
N3—C11	1.293 (3)	C10—H10B	0.9900
N3—C10	1.457 (3)	C11—C12	1.420 (3)
N3—H2N	0.90 (3)	C11—H11	0.9500
N4—C16	1.429 (3)	C12—C17	1.395 (3)
C1—C2	1.426 (3)	C12—C13	1.449 (3)
C1—C6	1.447 (3)	C13—C14	1.440 (3)
C2—C3	1.357 (3)	C14—C15	1.353 (3)
C2—H2	0.9500	C14—H14	0.9500
C3—C4	1.406 (3)	C15—C16	1.414 (3)
C3—H3	0.9500	C15—H15	0.9500
C4—C5	1.367 (3)	C16—C17	1.375 (3)
C5—C6	1.402 (3)	C17—H17	0.9500
			
O3—N1—O2	122.92 (18)	N2—C8—H8B	109.4
O3—N1—C4	118.46 (18)	C9—C8—H8B	109.4
O2—N1—C4	118.62 (18)	H8A—C8—H8B	108.0
C7—N2—C8	125.25 (19)	C8—C9—C10	114.79 (17)
C7—N2—H3N	110.9 (18)	C8—C9—H9A	108.6
C8—N2—H3N	123.8 (18)	C10—C9—H9A	108.6
C11—N3—C10	124.97 (18)	C8—C9—H9B	108.6
C11—N3—H2N	116.4 (16)	C10—C9—H9B	108.6
C10—N3—H2N	118.5 (16)	H9A—C9—H9B	107.5
O5—N4—O6	121.87 (18)	N3—C10—C9	111.32 (17)
O5—N4—C16	118.95 (18)	N3—C10—H10A	109.4
O6—N4—C16	119.19 (19)	C9—C10—H10A	109.4
O1—C1—C2	121.84 (18)	N3—C10—H10B	109.4
O1—C1—C6	121.70 (18)	C9—C10—H10B	109.4
C2—C1—C6	116.44 (18)	H10A—C10—H10B	108.0
C3—C2—C1	122.04 (19)	N3—C11—C12	124.13 (18)
C3—C2—H2	119.0	N3—C11—H11	117.9
C1—C2—H2	119.0	C12—C11—H11	117.9
C2—C3—C4	120.12 (19)	C17—C12—C11	118.54 (17)
C2—C3—H3	119.9	C17—C12—C13	120.74 (18)
C4—C3—H3	119.9	C11—C12—C13	120.70 (17)
C5—C4—C3	120.94 (19)	O4—C13—C14	121.84 (18)
C5—C4—N1	119.89 (19)	O4—C13—C12	121.81 (18)
C3—C4—N1	119.17 (18)	C14—C13—C12	116.34 (18)
C4—C5—C6	120.15 (18)	C15—C14—C13	121.78 (19)
C4—C5—H5	119.9	C15—C14—H14	119.1
C6—C5—H5	119.9	C13—C14—H14	119.1
C5—C6—C7	119.23 (18)	C14—C15—C16	120.17 (19)
C5—C6—C1	120.30 (18)	C14—C15—H15	119.9
C7—C6—C1	120.47 (18)	C16—C15—H15	119.9
N2—C7—C6	121.97 (19)	C17—C16—C15	121.02 (18)
N2—C7—H7	119.0	C17—C16—N4	119.63 (18)
C6—C7—H7	119.0	C15—C16—N4	119.33 (18)
N2—C8—C9	111.38 (17)	C16—C17—C12	119.93 (18)
N2—C8—H8A	109.4	C16—C17—H17	120.0
C9—C8—H8A	109.4	C12—C17—H17	120.0
			
O1—C1—C2—C3	−178.08 (18)	C11—N3—C10—C9	−118.0 (2)
C6—C1—C2—C3	0.2 (3)	C8—C9—C10—N3	69.0 (2)
C1—C2—C3—C4	−1.1 (3)	C10—N3—C11—C12	−178.09 (18)
C2—C3—C4—C5	0.9 (3)	N3—C11—C12—C17	−178.00 (18)
C2—C3—C4—N1	−179.06 (17)	N3—C11—C12—C13	0.3 (3)
O3—N1—C4—C5	174.81 (18)	C17—C12—C13—O4	179.94 (17)
O2—N1—C4—C5	−5.2 (3)	C11—C12—C13—O4	1.7 (3)
O3—N1—C4—C3	−5.2 (3)	C17—C12—C13—C14	−0.9 (3)
O2—N1—C4—C3	174.75 (18)	C11—C12—C13—C14	−179.17 (17)
C3—C4—C5—C6	0.2 (3)	O4—C13—C14—C15	−179.76 (19)
N1—C4—C5—C6	−179.88 (17)	C12—C13—C14—C15	1.1 (3)
C4—C5—C6—C7	177.95 (18)	C13—C14—C15—C16	−0.4 (3)
C4—C5—C6—C1	−1.0 (3)	C14—C15—C16—C17	−0.6 (3)
O1—C1—C6—C5	179.14 (18)	C14—C15—C16—N4	178.00 (18)
C2—C1—C6—C5	0.8 (3)	O5—N4—C16—C17	−4.5 (3)
O1—C1—C6—C7	0.2 (3)	O6—N4—C16—C17	175.25 (19)
C2—C1—C6—C7	−178.14 (17)	O5—N4—C16—C15	176.89 (18)
C8—N2—C7—C6	179.92 (17)	O6—N4—C16—C15	−3.4 (3)
C5—C6—C7—N2	−179.22 (18)	C15—C16—C17—C12	0.8 (3)
C1—C6—C7—N2	−0.2 (3)	N4—C16—C17—C12	−177.83 (17)
C7—N2—C8—C9	−128.3 (2)	C11—C12—C17—C16	178.29 (17)
N2—C8—C9—C10	69.6 (2)	C13—C12—C17—C16	0.0 (3)

### Hydrogen-bond geometry (Å, º)

**Table:**

*D*—H···*A*	*D*—H	H···*A*	*D*···*A*	*D*—H···*A*
N2—H3*N*···O1	0.93 (3)	1.77 (3)	2.583 (2)	145 (3)
N3—H2*N*···O4	0.90 (3)	1.93 (3)	2.642 (2)	135 (2)
N3—H2*N*···O4^i^	0.90 (3)	2.17 (3)	2.891 (2)	136 (2)

Symmetry code: (i) −*x*+1, −*y*, −*z*+1.
